# Comparing infectious risk of Trastuzumab-deruxtecan to Trastuzumab-emtansine in patients with breast cancer

**DOI:** 10.1007/s10549-026-07937-1

**Published:** 2026-03-07

**Authors:** Scott Gayfield, Jianing Ma, Michael Waleski, Joanne Kim, Daniel Stover, Margaret Gatti-Mays, Sachin R. Jhawar, Kai Johnson, Zeinab El Boghdadly, Kevin Ho

**Affiliations:** 1 Department of Internal Medicine, Ohio State University, Columbus, OH USA; 2 Center for Biostatistics, Ohio State University, Columbus, OH USA; 3 Department of Medicine, Ohio State University, Columbus, OH USA; 4 Department of Biomedical Informatics, Ohio State University, Columbus, OH USA; 5 Department of Oncology, Ohio State University, Columbus, OH USA; 6 Department of Radiation Oncology, Ohio State University, Columbus, OH USA; 7 Department of Infectious Disease, Ohio State University, Columbus, OH USA; 8 Department of Pulmonary, Critical Care, and Sleep Medicine, The Ohio State University Wexner Medical Center, 241 W 11 Ave, Suite 5000, Columbus, OH 43201 USA

**Keywords:** Trastuzumab-deruxtecan, Infection, Opportunistic, Lymphopenia

## Abstract

**Purpose:**

Trastuzumab-deruxtecan (T-DXd) is an antibody–drug conjugate (ADC) that revolutionized the treatment approach for breast cancer. However, the infectious risk associated with T-DXd is unknown. Here, we evaluate the infectious risk of T-DXd against trastuzumab-emtansine (T-DM1), an ADC with an identical monoclonal antibody.

**Methods:**

We conducted a retrospective study of consecutive breast cancer patients who received T-DXd or T-DM1. Demographic data, infection risk factors, infection sites, and opportunistic infections were recorded and compared across treatment groups. Multivariable logistic regression was used to evaluate the association between treatment group and infection, adjusting for clinical risk factors.

**Results:**

374 patients received T-DXd or T-DM1, with 126 receiving T-DXd alone, 196 receiving T-DM1 alone, and 52 patients receiving both treatments. Patients who received T-DXd did so as higher line of therapy (*p* < 0.001), was given more in the palliative setting (100% vs 33.7%, *p* < 0.001), had more prior immunosuppressive systemic treatment (78.6% vs 16.9%, *p* < 0.001), were exposed to more significant corticosteroid courses (17.2% vs 4.5%, *p* < 0.001), and had more hospitalizations during treatment (57.3% vs 27.7%, *p* < 0.001). Patients treated with T-DXd had a higher incidence of total infections (24.4% vs 14.0%, *p* = 0.01); in the infected population, unadjusted analysis reveals that those treated with T-DXd had more bloodstream infections (33.3% vs 5.9%, *p* = 0.004) and more infection-related mortality (18.2% vs 0%, *p* = 0.01). Three patients developed opportunistic infections on T-DXd, and 2 of the 3 were treated concurrently with high-dose corticosteroids. For multivariate analysis, after adjustment for clinically relevant variables and those associated with the outcome in univariate analyses, T-DXd was not associated with an increased risk of infection (OR = 1.89, 95% CI: 0.85–4.32, *p* = 0.12).

**Conclusion:**

Although patients receiving T-DXd had a higher incidence of infection, no significant difference in infectious risk was found after adjusting for several confounding variables. Infection-related mortality and opportunistic infections were rare and only occurred in the T-DXd cohort. Future prospective studies are warranted to more reliably evaluate the infectious risk of T-DXd compared to T-DM1, particularly as T-DXd is increasingly utilized earlier in the treatment course for breast cancer patients.

**Supplementary Information:**

The online version contains supplementary material available at 10.1007/s10549-026-07937-1.

## Introduction

Trastuzumab-deruxtecan (T-DXd) is an antibody–drug conjugate consisting of trastuzumab, a monoclonal antibody targeting human epidermal growth factor receptor 2 (HER2), conjugated with deruxtecan, a cytotoxic topoisomerase I inhibitor, to target HER2-expressing cancer cells to enhance therapeutic efficacy. Use of T-DXd has revolutionized the treatment approach for patients with both HER-2 positive and HER-2 low metastatic breast cancer, leading to longer progression-free and overall survival compared to previous standard of care chemotherapy and targeted therapy [1–3].

While pneumonitis and interstitial lung disease are the most concerning complications of T-DXd treatment [4, 5], little is known about the infection risk associated with T-DXd in breast cancer patients. Pooled analyses of existing studies outlined different types of infections linked to T-DXd [6], and small studies suggested increased risk of bacterial and *Pneumocystis jiroveci* pneumonia (PJP) with T-DXd compared to trastuzumab-emtansine (T-DM1) [7], but the context of these infections (line of therapy, systemic corticosteroid use, concurrent neutropenia or lymphopenia) is unknown.

To address this knowledge gap, we conducted a single-center retrospective analysis of breast cancer patients treated with T-DXd and T-DM1, which has an identical monoclonal antibody, to assess the infectious risk of T-DXd compared to another commonly utilized antibody–drug conjugate for breast cancer patients.

## Methods

### Data collection, included patients

We conducted a retrospective study of consecutive patients with breast cancer who received at least one dose of T-DXd or T-DM1 at The Ohio State University (OSU) from January 1, 2019, to February 20, 2024. Study data were compiled using REDCap electronic data capture tools at OSU [8, 9]. The study protocol was reviewed and approved by The OSU Institutional Review Board (2023C0104), and a waiver of informed consent was granted due to the retrospective nature of the study.

### Demographic and infectious variables

The following potential risk factors for infection were collected: age, body mass index, smoking status, history of cirrhosis, history of diabetes, history of chronic kidney disease, history of chronic lung diseases (chronic obstructive pulmonary disease, asthma, interstitial lung disease), history of autoimmune disease (all were recorded except Hashimoto’s thyroiditis), number of hospitalizations while on treatment, presence of central line during treatment, line of therapy, lymphocyte and neutrophil count at T-DM1 or T-DXd treatment initiation and at time of infection, and concurrent significant systemic corticosteroid exposure at time of T-DM1 or T-DXd treatment and at the time of infection. We defined significant corticosteroid exposure as prednisone equivalent of 20 mg daily for at least 7 consecutive days to exclude intermittent corticosteroid administration (steroid given as pre-medication during T-DM1 or T-DXd therapy, short courses for respiratory illness, etc.) that is not as reflective of the immunosuppressive effect of more prolonged steroid exposure.

### Infectious sties

Infections were recorded in the following fashion: 1). Bloodstream 2). Respiratory 3). Skin/soft tissue 4). Urine 5). Gastrointestinal 6). Abdominal. Infections were documented by the clinical team at the time of infection and confirmed by the study team retrospectively. Bloodstream infections were defined by the presence of bacterial or fungal microorganisms in the bloodstream that were considered pathogenic [10]. Respiratory infections were recorded if there was both a pathogenic bacterial or fungal organism recovered on lower respiratory culture (sputum, tracheal aspirate, bronchoalveolar lavage) or a positive antigen/PCR test along with clinical signs of respiratory infection (clinical symptoms [fever, malaise, cough, hemoptysis], signs of infection on chest imaging). Viral respiratory infections were recorded only if patients had persistently positive testing and symptoms (positive antigen/PCR and persistent respiratory symptoms over at least 21 days) suggestive of poor viral clearance as this is more reflective of impaired immunity/infection risk compared to recording single incidence of antigen/PCR positivity [11, 12]. Skin and soft tissue infections were recorded if antimicrobial treatment was initiated based on the skin and soft tissue findings [13]. Urinary infections were recorded if there was a positive urine culture and the patient had clinical symptoms of infection (systemic or focused urinary symptoms) [14]. We excluded patients who were empirically treated for urinary infections without a positive culture. Gastrointestinal infections were recorded with a positive infectious stool test (culture, antigen, PCR) along with clinical symptoms necessitating targeted treatment. Abdominal infections were recorded for clinical symptoms of abdominal infection along with suggestive imaging findings (included cholecystitis, diverticulitis, pancreatitis, peritonitis). Opportunistic infections (PJP, endemic mycoses, mold, nocardia, legionella, atypical mycobacterium, cytomegalovirus [CMV]) were captured separately. CTCAE scoring was utilized to define infection severity, with mild infections defined as grade 1–2 and severe infections as grade 3–5 [15].

### Timing of infection

Infection was attributed to either T-DXd or T-DM1 if the infection was recorded during time on treatment or in the 2 months following treatment discontinuation. Delays in administration of T-DXd or T-DM1 or permanent discontinuation of therapy following infections were also recorded.

### Statistical analysis

The primary endpoint was infection during treatment, and the objective was to evaluate the association between treatment groups (T-DXd vs. T-DM1) and infection, severe infections, and infection-related mortality. Patients could have received either T-DXd, T-DM1, or both; for those who received both, only the first treatment was considered in the primary analysis. Baseline characteristics and risk factors were compared between patients receiving T-DXd and those receiving T-DM1. Categorical variables were summarized as counts and percentages, while continuous variables were summarized as means with standard deviations (SD) for normally distributed variables or medians with interquartile ranges (IQR, 25th–75th percentile) for non-normally distributed variables. Multivariable logistic regression was used to examine the association between treatment group and infection, adjusting for relevant covariates. Covariates were selected based on clinical relevance, with additional consideration given to variables that showed an association in univariate analysis, they are include: line of therapy (1st vs others), lymphocyte count and neutrophil count at time of infection, diabetes, cirrhosis, chronic lung disease (chronic obstructive pulmonary disease, asthma, interstitial lung disease), systemic treatment immediately prior to T-DXd to T-DM1 therapy (immunosuppresive agents vs others), age, and race (white vs others). P-values from univariate analyses were unadjusted, and observations with missing values in the variables of interest were excluded. All analyses were conducted using R software (version 4.5.0; R Foundation for Statistical Computing, Vienna, Austria).

## Results

**Fig. 1:**
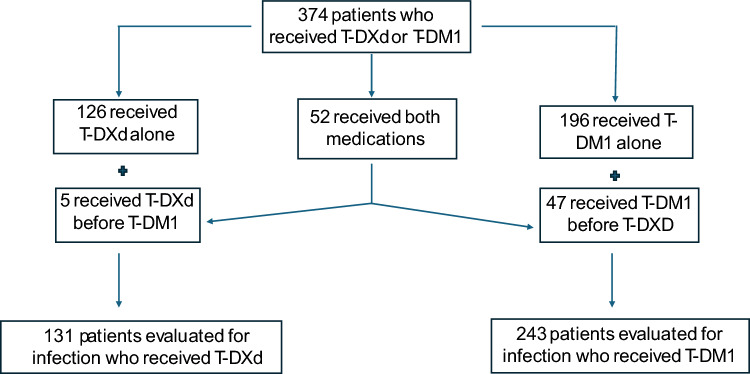
Flowchart of included patients

The following flowchart summarizes included patients, number of patients who received T-DM1, number of patients who received T-DXd, and those that received both treatments (Fig. 1).

### Demographics

We identified 374 patients who received either T-DXd or T-DM1. Of the 374 patients, 52 received both treatments, 126 received T-DXd alone, and 196 received T-DM1 alone. Of the 52 patients who received both treatments, 5 received T-DXd first, while 47 received T-DM1 first. Patients receiving T-DXd were treated more commonly in the palliative setting (*p* < 0.001), as higher line of therapy (*p* < 0.001), exposed more frequently to significant corticosteroids (17.2% vs 4.5%, *p* < 0.001), had more immunosuppressive prior systemic treatment (78.6% vs 16.9%, *p* < 0.001), had lower lymphocyte count at start of treatment (1110 [730, 1,550] vs 1,460 [1110, 1,900] cells/µL, *p* < 001), and had higher incidence of hospitalizations during treatment (57.3% vs 27.7%, *p* < 0.001) compared to those treated with T-DM1. There were no other significant differences in demographics or relevant medical history between patients who received T-DM1 and T-DXd (Table 1). Of the patients who received T-DXd, 23 patients (12.8%) developed T-DXd pneumonitis.

**Table 1:** Demographic differences between patients who received T-DM1 versus T-DXd

Variable	T-DM1 (*n* = 243)	T-DXd (*n* = 131)	*p*-value
Age	54 (44, 64)	57 (46, 66)	0.13
BMI	28 (23, 33)	26 (23, 31)	0.10
History of smoking	80 (32.9%)	45 (34.3%)	0.78
Diabetes	37 (15.2%)	28 (20.6%)	0.13
Cirrhosis	20 (8.2%)	19 (14.5%)	0.06
Chronic kidney disease	16 (6.6%)	10 (7.6%)	0.70
Chronic pulmonary diseases^1^	35 (14.4%)	23 (17.6%)	0.42
Autoimmune diseases^2^	4 (1.6%)	3 (2.3%)	0.70
Line of therapy			< 0.001
1	172 (70.8%)	5 (3.8%)	
2 or higher	71 (29.2%)	126 (96.2%)	
Curative intent	161 (66.3%)	0 (0%)	< 0.001
Significant corticosteroid exposure^3^	9 (4.5%)	22 (17.2%)	< 0.001
Incidence of hospitalization	67 (27.7%)	75 (57.3%)	< 0.001
Central line	201 (82.7%)	105 (80.2%)	0.49
Lymphocyte count at treatment start (cells/µL)	1,460 (1,110, 1,900)	1,100 (730, 1,550)	< 0.001
Neutrophil count at treatment start (cells/µL)	3,420 (2,450, 4,540)	3,740 (2,510, 5,980)	0.12
Prior systemic treatment			< 0.001
Less immunosuppressive^4^	202 (83.1%)	28 (21.4%)	
More immunosuppressive^5^	41 (16.9%)	103 (78.6%)	

^1^Chronic pulmonary disease: Includes chronic obstructive pulmonary disease, asthma, and interstitial lung disease

^2^Includes multiple sclerosis, Grave’s disease, pernicious anemia, autoimmune hepatitis, Sjogren’s syndrome, and mixed connective tissue disorder

^3^Steroid equivalent of prednisone 20 mg for at least 7 days of consecutive treatment

^4^Includes immunotherapy, targeted therapies, no prior treatment

^5^Includes cytotoxic chemotherapy, cyclin-dependent kinase 4/6 inhibitors, antibody–drug conjugates, PIK3CA inhibitors, PARP inhibitors

### Infected population

Thirty-four of the 243 patients who received T-DM1 developed infections, and 32 of the 131 patients developed infections while on T-DXd. Time on treatment was similar between the two groups (287 days [220, 364] for T-DM1, 196 days [90, 464] for T-DXd). T-DXd was more commonly utilized as 2nd line or higher treatment and for palliative intent compared to T-DM1 (*p* < 0.001 for both). Of those with infections, patients treated with T-DXd had lower lymphocyte count at the time of infection (790 cells/µL [340, 1,120] vs 1,295 cells/µL [840, 1,975], *p* = 0.002), more severe infections (59.4% vs 29.4%, *p* = 0.01), and more delays or permanent discontinuations of treatment following infection (62.5% vs 21.2%, *p* < 0.001) compared to T-DM1. Six patients (19.4%) treated with T-DXd had significant steroid exposure during their treatment course, while 1 (3.4%) patient had significant steroid exposure while on T-DM1. There was no significant difference in time to first infection, baseline lymphocyte and neutrophil counts, and neutrophil count at the time of infection between the treatment groups (Table 2).

**Table 2:** Clinical characteristics of infected population

	T-DM1 (*n* = 34)	T-DXd (*n* = 32)	*P*-value
Time to 1 st infection (days)	108 (50, 275)	114 (37, 227)	0.72
Line of therapy			< 0.001
1	20 (58.8%)	3 (9.4%)	
2 or higher	14 (41.2%)	29 (90.6%)	
Curative intent	19 (55.9%)	0 (0%)	< 0.001
Significant corticosteroid exposure during treatment^1^	1 (3.4%)^2^	6 (19.4%)^3^	
Prior systemic treatment			< 0.001
Less immunosuppressive^4^	28 (82.4%)	9 (28.1%)	
More immunosuppressive^5^	6 (17.6%)	23 (71.9%)	
Lymphocyte count at treatment initiation (cells/µL); *median (range)*	1,420 (1,010, 1,910)	1,230 (880, 1,600)	0.12
Neutrophil count at treatment initiation (cells/µL); *median (range)*	3,330 (2,500, 4,510)	4,065 (2,825, 6,295)	0.21
Lymphocyte count at time of infection (cells/µL); *median (range)*	1,295 (840, 1,975)	790 (340, 1,120)	0.002
Neutrophil count at time of infection (cells/µL); *median (range)*	4,050 (2,800, 5,630)	2,950 (1,050, 7,360)	0.32
Severe infections^6^	10 (29.4%)	19 (59.4%)	0.01
Delay or permanent discontinuation following infection	7 (21.2%)	20 (62.5%)	< 0.001

^1^Steroid equivalent of prednisone 20 mg for at least 7 days of consecutive treatment. No p-value calculated due to small numbers in each group

^2^On corticosteroids for neurological symptoms related to brain radiation

^3^Five patients were on steroids for drug-related pneumonitis, one for neurologic symptoms related to brain radiation

^4^Includes immunotherapy, targeted therapies, no prior treatment

^5^Includes cytotoxic chemotherapy, cyclin-dependent kinase 4/6 inhibitors, antibody–drug conjugates, PIK3CA inhibitors, PARP inhibitors

^6^Severe infections defined as CTCAE grade 3 or higher events

### Infection incidence and types

Patients treated with T-DXd had a higher incidence of total infections compared to those treated with T-DM1 (24.4% vs 14.0%, *p* = 0.01). From the infected population, patients treated with T-DXd had higher incidence of bloodstream infections (34.3% vs 5.9%, *p* = 0.004) and infection-related mortality (18.8% vs 0%, *p* = 0.01). Patients treated with T-DM1 had higher incidence of skin and soft tissue infections (23.5% vs 3.1%, *p* = 0.03). One of the 6 patients who developed respiratory infection had concurrent T-DXd pneumonitis. No other differences in infection locations were observed between the treatment groups (Table 3). Three patients developed PJP pneumonia while receiving T-DXd treatment, and 1 of the 3 had concurrent CMV pneumonitis. Of the 3 patients who developed opportunistic infections, two were receiving high-dose steroids (> 1 mg/kg for 15 and 18 days at time of infection) for pneumonitis (1 attributed to immune checkpoint inhibitor [ICI], 1 from T-DXd]). Lymphocyte count at time of infection was 360, 320, and 130 cells/µL and none were on PJP prophylaxis. No opportunistic infections were identified in the T-DM1 treatment group.

**Table 3:** Incidence and Types of Infection while on T-DXd and T-DM1 Treatment

All patients	T-DXd (*n* = 131)	T-DM1 (*n* = 243)	*p*-value
Overall infection incidence	32 (24.4%)	34 (14.0%)	0.01
Infected patients	**T-DXd (*n* = 32)**	**T-DM1 (*n* = 34)**	***p*-value**
Infection-related Mortality^1^	6 (18.8%)	0 (0%)	0.01
Bloodstream infections^2^	11 (34.3%)	2 (5.9%)	0.004
Respiratory infections^3^	6 (18.8%)	1 (2.9%)	0.051
Skin/Soft tissue	1 (3.1%)	8 (23.5%)	0.03
Urinary infections	20 (62.5%)	25 (73.5%)	0.34
Gastrointestinal	1 (3.1%)	0 (0%)	0.49
Abdominal	1 (3.1%)	0 (0%)	0.49
Opportunistic infections^4^	3 (9.4%)	0 (0%)	0.11

^1^Two had respiratory infections, 2 had bloodstream infections, and 2 had both respiratory and bloodstream infections. Two of the 4 respiratory events resulting in death were from *Pneumocystis Jiroveci*

^2^Both patients who had bloodstream infections (BSI) in the T-DM1 group had a central line at the time of infection and were attributed to the central line (CLABSI). For T-DXd, 9 of the 11 patients who had BSI had a central line at the time of infection, and 2 of the 9 were attributed to CLABSI

^3^ T-DM1: *Moraxella catarrhalis*; T-DXd: *Stenotrophomonas maltophilia* and *Pneumocystis jiroveci, Pseudomonas aeruginosa* and *Serratia marcescens*, *Pneumocystis Jiroveci* and *Cytomegalovirus*, *Staphylococcus Aureus*, *Streptococcus pneumoniae*, *Pneumocystis Jiroveci, COVID-19 (persistent positivity over 6 weeks with clinical respiratory symptoms)*

^4^One patient had both *Pneumocystis jiroveci* and *Cytomegalovirus*. Two patients had *Pneumocystis jiroveci* infection without another concurrent opportunistic infection

### Comparison of T-DXd and T-DM1 in the palliative setting

There was no difference in overall incidence of infection between those treated with T-DXd and T-DM1 in the palliative setting (24.4% vs 18.3%, *p* = 0.29). In the infected cohort, patients treated with T-DXd had more severe infections (59.4% vs 13.3%, *p* = 0.003), more delays and discontinuations in treatment (62.5% vs 7.1%, *p* < 0.001), and lower lymphocyte count at time of infection (790 [340, 1,120] cells/µL vs 1,180 [630, 1,790] cells/µL, *p* = 0.05) compared to those treated with T-DM1. Patients treated with T-DXd had a higher rate of bloodstream infections (34.3% vs 6.7%, *p* = 0.07) and infection-related mortality (18.8% vs 0%, p = 0.16), but these were not statistically significant (Supplementary Table 1).

### Risk factors for infection-related mortality and severe infections from T-DXd

No variables, including lymphocyte and neutrophil count at the time of infection (260 vs 1000 cells/µL, *p* = 0.07, 985 vs 3,130 cells/µL, *p* = 0.12, respectively), were associated with increased risk of death when comparing patients who developed infections while on T-DXd therapy. One of the 6 patients who died from infection were exposed to significant corticosteroids at the time of infection (Supplementary Table 2). Patients treated with T-DXd who developed severe infections had lower lymphocyte count (455 [150, 1,000] cells/µL vs 1,080 [790, 1,280] cells/µL, *p* = 0.005) at the time of infection compared to mild infections, and no other significant associations were identified (Supplementary Table 3).

### Multivariate analyses of infectious risk of T-DXd and T-DM1

A total of 369 patients were included in the final multivariable model. Fewer than 5% of observations had missing data and were excluded using a complete-case approach. Specifically, five patients had missing values for lymphocyte and neutrophil counts at the time of infection and were therefore removed from the final analysis. After adjusting for line of therapy, prior systemic treatment, lymphocyte count and neutrophil count at time of infection, relevant comorbidities, age, and race, patients who received T-DXd treatment had higher odds of infection compared to those who received TDM1 (OR = 1.89, 95% CI: 0.85–4.32, *p* = 0.12), but this difference is not statistically significant (Table 4).

**Table 4:** Multivariate analysis evaluating the infectious risk of T-DXd vs T-DM1

Variable	Odds Ratio	95% CI	*p*-value
Treatment group			
T-DM1			
T-DXd	1.89	0.85–4.32	0.12
Line of therapy			
1			
2 or higher	1.64	0.72–3.64	0.23
Lymphocyte count at time of infection^1^	0.98	0.72–1.20	0.87
Neutrophil count at time of infection^1^	1.01	0.91–1.11	0.87
Age	0.99	0.96–1.01	0.23
Race			
White			
Other races	0.35	0.11–0.87	0.04
Diabetes	1.15	0.52–2.43	0.72
Cirrhosis	0.78	0.27–1.95	0.62
Chronic lung disease^2^	1.42	0.64–3.00	0.37
Prior systemic treatment			
Less immunosuppressive^3^			
More immunosuppressive^4^	0.67	0.31–1.41	0.30

^1^Lymphocyte and neutrophil count at time of infection treated as a continuous variable. Model coefficients and odds ratios correspond to the change in infection risk associated with a 1000-unit increase in each cell count instead of 1 unit increase to improve interpretability

^2^Includes chronic obstructive pulmonary disease, asthma, and interstitial lung disease

^3^Includes immunotherapy, targeted therapies, no prior treatment

^4^Includes cytotoxic chemotherapy, cyclin-dependent kinase 4/6 inhibitors, antibody–drug conjugates, PIK3CA inhibitors, PARP inhibitors

## Discussion

In this single-center, retrospective cohort study of consecutive breast cancer patients who received either T-DM1 or T-DXd, we found that incidence of total infections, severe infections, and bloodstream infections were higher in patients treated with T-DXd, and all infection-related mortality occurred in the T-DXd treatment group. After adjusting for selected confounders, we found no significant association with T-DXd and infection risk compared to treatment with T-DM1. To our knowledge, this is the first comprehensive study to evaluate the infectious risk of T-DXd compared to another antibody–drug conjugate utilized for breast cancer patients.

Landmark studies examining the efficacy of T-DXd compared to T-DM1 in metastatic HER2 + breast cancer patients did not report on the infectious risk between treatment groups [16], and prior studies focused on the infection risk of T-DXd used the FDA Adverse Event Reporting System (FAERS), a database that collects adverse events reported to the FDA but provides limited real-world context regarding the circumstances in which these events occurred [6, 7]. While studies utilizing FAERS describe lung infections associated with T-DXd treatment, including a higher risk of infection compared to T-DM1 [7], it is unclear whether these events represented true infection or non-infectious pneumonitis that were empirically treated with antimicrobials. Pertinent co-morbidities, concurrent immunosuppressive medications, and lab values that may increase the risk of infection are also unknown. In our study, we included only patients with symptomatic, culture or imaging-confirmed infections to ensure that recorded events were truly infectious. Though incidence of infection was higher for T-DXd, we did not find a significant association between infection risk and T-DXd compared to T-DM1 after adjusting for key confounding variables. In unadjusted analysis looking only at those treated in the palliative setting, we also did not see a difference in overall infection incidence between T-DXd and T-DM1. This suggests the increased incidence of infection for patients treated with T-DXd may be related to concurrent medical illness rather than just the drug itself. As most of our patients received T-DXd as 2nd line or higher therapy and were treated with palliative intent, larger studies are needed to fully investigate the infectious risk of T-DXd as it is increasingly replacing T-DM1 earlier in the breast cancer treatment course in the curative and adjuvant settings [17].

While opportunistic infections were rare in both treatment groups, patients treated with T-DXd had a higher incidence of atypical infections. Two patients treated with T-DXd developed PJP and 1 developed both PJP and CMV pneumonitis while receiving corticosteroid treatment for T-DXd pneumonitis. Previous reports describe PJP infections with T-DXd treatment [6, 7], and others list opportunistic infection events (CMV, Aspergillus, and PJP) [18]. However, none of these examined whether these infections occurred with concurrent corticosteroid use or lymphopenia, which are major risk factors associated with PJP and other atypical infections in non-HIV populations [19, 20]. Based on our study, opportunistic infections may be related to the concurrent use of T-DXd and high-dose (≥ 1 mg/kg) corticosteroids and/or severe lymphopenia (< 500 cells/µL) and not directly related to T-DXd alone; though T-DXd can cause lymphopenia [21], only one of the T-DXd treated patients developed opportunistic infections without concurrent high-dose steroid use, and all three patients had severe lymphopenia at the time of infection. Clinicians should strongly consider PJP prophylaxis for patients treated with T-DXd when initiating long courses of high-dose corticosteroids and/or in those with severe lymphopenia.

The higher incidence of bloodstream infections in the T-DXd group may reflect nosocomial factors rather than a direct drug effect. In our study, patients treated with T-DXd had more hospitalizations (possibly due to more advanced disease or later-line therapy) compared to those treated with T-DM1, and bloodstream infections are commonly associated with frequent hospitalizations [22]. Infection-related mortality was more frequent in the T-DXd group with no infection-related deaths in patients who received T-DM1, and those with severe infections had lower lymphocyte count at the time of infection compared to those with mild infections when treated with T-DXd. Of the six patients who died during T-DXd treatment, two of the four fatal respiratory infections were PJP occurring in the context of severe lymphopenia. One was also on high-dose corticosteroids for drug-related pneumonitis, another well-established risk factor for both lymphopenia and PJP. Although neutrophil counts before treatment and at infection onset did not differ significantly between groups, two patients who died from bloodstream infections had severe neutropenia (< 500 cells/µL) at infection. Given the relatively small number of severe infections and infection-related deaths in our study, further studies are needed to assess the impact of concurrent comorbidities, lymphocytic and neutrophilic functions, and T-DXd-related immunosuppression in the development of severe infections.

There are several limitations to our study. First, as a single-center retrospective study at a tertiary referral center, results may not apply to all breast cancer patients treated with T-DXd. Unrecognized confounders could affect the observed relationship between T-DXd, T-DM1, and infection risk. Additionally, many of our patients received T-DXd with palliative intent, making it difficult to discern the true impact of the drug on infection risk compared to patients who received T-DM1 for curative intent. We addressed this by adjusting for line of therapy, prior systemic therapy, and lymphocyte and neutrophil count at time of infection. To ensure accurate infection assessment, we only included patients with definitive evidence of infection (positive culture/PCR) to objectively evaluate the infectious risk of T-DXd. By doing so, we may have excluded a subset of patients with culture-negative infections. However, our infection numbers are in line with prior studies examining infections related to T-DXd [6, 7, 10], suggesting generalizability of our results.

## Conclusion

Although T-DXd was associated with a higher incidence of infection compared to T-DM1, no significant difference in infectious risk was found after adjusting for several confounding variables. Infection-related mortality and opportunistic infections were rare and occurred only within the T-DXd cohort. Future prospective studies are warranted to more reliably evaluate the infectious risk of T-DXd compared to T-DM1, particularly as T-DXd is increasingly utilized earlier in the treatment course for breast cancer patients.

## Supplementary Information

Below is the link to the electronic supplementary material.

Supplementary file1 (DOCX 21 KB)

Supplementary file2 (DOCX 16 KB)

Supplementary file3 (DOCX 17 KB)

## Data Availability

The datasets used and/or analyzed during the current study are available from the corresponding author on reasonable request.

## References

[1] Modi S, Saura C, Yamashita T et al (2020) Trastuzumab Deruxtecan in previously treated HER2-Positive breast cancer. N Engl J Med 382(7):610–621 31825192 10.1056/NEJMoa1914510 PMC7458671

[2] Modi S, Jacot W, Yamashita, et al. Trastuzumab Deruxtecan in previously treated HER2-Low advanced breast Cancer. N Engl J Med. 2022;387(1):9–20. 10.1056/NEJMoa2203690 PMC10561652 35665782

[3] Hurvitz SA, Hegg R, Chung WP et al (2023) Trastuzumab deruxtecan versus Trastuzumab Emtansine in patients with HER2-positive metastatic breast cancer: updated results from DESTINY-Breast03, a randomised, open-label, phase 3 trial. Lancet 401(10371):105–117 36495879 10.1016/S0140-6736(22)02420-5

[4] Powell CA, Modi S, Iwata H et al (2022) Pooled analysis of drug-related interstitial lung disease and/or pneumonitis in nine Trastuzumab Deruxtecan monotherapy studies. ESMO Open 7(4):100554 35963179 10.1016/j.esmoop.2022.100554 PMC9434416

[5] Henricks J, Haddad T, Ahmed O et al (2025) Evaluating risk factors for Trastuzumab-Deruxtecan Pneumonitis in patients with metastatic breast cancer. Breast Cancer Res 27(1):16 39901280 10.1186/s13058-025-01967-1 PMC11792225

[6] Guo Z, Ding Y, Wang M et al (2022) Safety of Trastuzumab Deruxtecan: a meta-analysis and pharmacovigilance study. J Clin Pharm Ther 47(11):1837–1844 36200429 10.1111/jcpt.13777 PMC9827941

[7] Ma P, Tian H, Shi Q et al (2023) High risks adverse events associated with trastuzumab emtansine and trastuzumab deruxtecan for the treatment of HER2-positive/mutated malignancies: a pharmacovigilance study based on the FAERS database. Expert Opin Drug Saf 22(8):685–696 37068935 10.1080/14740338.2023.2204228

[8] Harris PA, Taylor R, Thielke R et al (2009) Research electronic data capture (REDCap) **– **a metadata-driven methodology and workflow process for providing translational research informatics support. J Biomed Inform 42(2):377–381 18929686 10.1016/j.jbi.2008.08.010 PMC2700030

[9] PA Harris, R Taylor, BL Minor et al (2019) REDCap Consortium, The REDCap consortium: Building an international community of software partners. J Biomed Inform. 10.1016/j.jbi.2019.103208 PMC7254481 31078660

[10] Viscoli C (2016) Bloodstream Infections: The peak of the iceberg. Virulence 7(3):248–251 26890622 10.1080/21505594.2016.1152440 PMC4871637

[11] Lyudovyk O, Kim JY, Qualls D, Hwee MA, Lin YH, Boutemine SR, Elhanati Y, Solovyov A, Douglas M, Chen E, Babady NE, Ramanathan L, Vedantam P, Bandlamudi C, Gouma S, Wong P, Hensley SE, Greenbaum B, Huang AC, Vardhana SA (2022) Impaired humoral immunity is associated with prolonged COVID-19 despite robust CD8 T cell responses. Cancer Cell 40(7):738-753.e5 35679859 10.1016/j.ccell.2022.05.013 PMC9149241

[12] Psaros Einberg A, Casswall TH, Arnell H, Nowak G, Mirazimi A, Sundin M, Fischler B (2021) Iatrogenic immunosuppression can lead to prolonged viral shedding and absent immune response to COVID-19. Acta Paediatr 110(10):2810–2811 34043855 10.1111/apa.15955 PMC8222928

[13] Stevens DL, Bisno AL, Chambers HF et al (2014) Practice guidelines for the diagnosis and management of skin and soft tissue infections: 2014 update by the Infectious diseases society of America. Clin Infect Dis 59(2):e10-52 24973422 10.1093/cid/ciu444

[14] Trautner BW, Cortés-Penfield NW, Gupta K, Hirsch EB, Horstman M, Moran GJ, Colgan R, O'Horo JC, Ashraf MS, Connolly S, Drekonja D, Grigoryan L, Huttner A, Lazenby GB, Nicolle L, Schaeffer A, Yawetz S, Lavergne V (2025) Clinical Practice Guideline by Infectious Diseases Society of America (IDSA): 2025 Guideline on Management and Treatment of Complicated Urinary Tract Infections: Timing of Intravenous to Oral Antibiotics Transition for Complicated UTI. Clin Infect Dis 19:ciaf461. 10.1093/cid/ciaf461 41414903

[15] Freites-Martinez A, Santana N, Arias-Santiago S, Viera A (2021) Using the common terminology criteria for adverse events (CTCAE - Version 5.0) to evaluate the severity of adverse events of anticancer therapies. Actas Dermosifiliogr (Engl Ed) 112(1):90–92 32891586 10.1016/j.ad.2019.05.009

[16] Cortés J, Kim SB, Chung WP, Im SA, Park YH, Hegg R, Kim MH, Tseng LM, Petry V, Chung CF, Iwata H, Hamilton E, Curigliano G, Xu B, Huang CS, Kim JH, Chiu JWY, Pedrini JL, Lee C, Liu Y, Cathcart J, Bako E, Verma S, Hurvitz SA (2022) Destiny-breast03 trial investigators trastuzumab deruxtecan versus trastuzumab emtansine for breast cancer. N Engl J Med 386(12):1143–1154 35320644 10.1056/NEJMoa2115022

[17] Geyer CE, Park YH, Shao Z-M, Huang C-S, Barrios CHE, Abraham J, Prat A, Niikura N, Untch M, Im S-A, Li W, Li H, Wang Y, Yao H, Kim S-B, Mathias E, Sato Y, Lu W, Abdel-Monem H, Loibl S (2025) LBA1 Trastuzumab deruxtecan (T-DXd) vs trastuzumab emtansine (T-DM1) in patients (pts) with high-risk human epidermal growth factor receptor 2–positive (HER2+) primary breast cancer (BC) with residual invasive disease after neoadjuvant therapy (tx): interim analysis of DESTINY-Breast05. Ann Oncol 36:S1671–S1672

[18] Harbeck N, Ciruelos E, Jerusalem G, Müller V, Niikura N, Viale G, Bartsch R, Kurzeder C, Higgins MJ, Connolly RM et al (2024) Trastuzumab deruxtecan in HER2-positive advanced breast cancer with or without brain metastases: a phase 3b/4 trial. Nat Med 30(12):3717–3727 39271844 10.1038/s41591-024-03261-7 PMC11645283

[19] Quigley N, d’Amours L, Gervais P, Dion G (2023) Epidemiology, risk factors, and prophylaxis use for pneumocystis jirovecii pneumonia in the non-HIV population: a retrospective study in Québec Canada. Open Forum Infect Dis 11(1):639 10.1093/ofid/ofad639 PMC10810061 38274551

[20] Sowden E, Carmichael AJ (2004) Autoimmune inflammatory disorders, systemic corticosteroids and pneumocystis pneumonia: a strategy for prevention. BMC Infect Dis 16(4):42 10.1186/1471-2334-4-42 PMC526257 15488151

[21] Jerusalem G, Park YH, Yamashita T et al (2022) Trastuzumab deruxtecan in HER2-positive metastatic breast cancer patients with brain metastases: A destiny-breast01 subgroup analysis. Cancer Discov 12(12):2754–2762 36255231 10.1158/2159-8290.CD-22-0837 PMC9716244

[22] Diekema DJ, Beekmann SE, Chapin KC, Morel KA, Munson E, Doern GV (2003) Epidemiology and outcome of nosocomial and community-onset bloodstream infection. J Clin Microbiol 41(8):3655–3660 12904371 10.1128/JCM.41.8.3655-3660.2003 PMC179863

