# Supplementary material for: Comparing infectious risk of Trastuzumab-deruxtecan to Trastuzumab-emtansine in patients with breast cancer
Source: Breast Cancer Res Treat. 2026 Mar 7;216(2):23. doi: 10.1007/s10549-026-07937-1 (PMC12967492; doi:10.1007/s10549-026-07937-1)
Supplement: Supplementary file 1 — Supplementary file1 (DOCX 21 KB) [file 10549_2026_7937_MOESM1_ESM.docx]

Supplementary Table 1: Incidence and Types of Infection while on T-DXd and T-DM1 Treatment in the Palliative Setting

| **All patients** | **T-DXd (n=131)** | **T-DM1 (n=82)** | **p-value** |
| --- | --- | --- | --- |
| Overall Infection Incidence | 32 (24.4%) | 15 (18.3%) | 0.29 |
| **Infected patients** | **T-DXd (n=32)** | **T-DM1 (n=15)** | **p-value** |
| Line of therapy |  |  | 1.00 |
| 1 | 3 (9.4%) | 1 (6.7%) |  |
| 2 or higher | 29 (90.6%) | 14 (93.3%) |  |
| Significant Steroid Exposure During Treatment^1^ | 6 (19.4%) | 1 (7.1%) |  |
| Lymphocyte Count at Treatment Initiation (cells/µL) | 1,230 (880, 1,615) | 1,015 (700, 1,710) | 0.56 |
| Neutrophil Count at Treatment Initiation (cells/µL) | 4,065 (2.825, 6,295) | 2,910 (2,310, 4,000) | 0.12 |
| Lymphocyte Count at Time of Infection (cells/µL) | 790 (340, 1,120) | 1,180 (630, 1,790) | 0.05 |
| Neutrophil Count at Time of Infection (cells/µL) | 2,950 (1,050, 7,360) | 3,510 (2,160, 4,370) | 0.72 |
| Severity^2^ |  |  | 0.003 |
| Mild | 13 (40.6%) | 13 (86.7%) |  |
| Severe | 19 (59.4%) | 2 (13.3%) |  |
| Delays or Discontinuation | 20 (62.5%) | 1 (7.1%) | <0.001 |
| Infection-related Mortality^3^ | 6 (18.8%) | 0 (0%) | 0.16 |
| Location of Infection |  |  |  |
| Bloodstream infections | 11 (34.3%) | 1 (6.7%) | 0.07 |
| Respiratory infections^4^ | 6 (18.8%) | 1 (6.7%) | 0.40 |
| Skin/Soft Tissue | 1 (3.1%) | 0 (0%) | 1.00 |
| Urinary infections | 20 (62.5%) | 14 (93.3%) | 0.04 |
| Gastrointestinal | 1 (3.1%) | 0 (0%) | 1.00 |
| Abdominal | 1 (3.1%) | 0 (0%) | 1.00 |

^1^ Steroid equivalent of prednisone 20 mg for at least 7 days of consecutive treatment. No p-value calculated due to small numbers in each group

^2^Severe infections defined as CTCAE grade 3 or higher events

^3^Two had respiratory infections, 2 had bloodstream infections, and 2 had both respiratory and bloodstream infections. Two of the 4 respiratory events resulting in death were from *Pneumocystis Jiroveci*

^4^T-DM1: *Moraxella catarrhalis*; T-DXd: *Stenotrophomonas maltophilia* and *Pneumocystis jiroveci, Pseudomonas aeruginosa* and *Serratia marcescens*, *Pneumocystis Jiroveci* and *Cytomegalovirus*, *Staphylococcus Aureus*, *Streptococcus pneumoniae*, *Pneumocystis Jiroveci, COVID-19 (persistent positivity over 6 weeks with clinical respiratory symptoms)*
