# Supplementary material for: Comparing infectious risk of Trastuzumab-deruxtecan to Trastuzumab-emtansine in patients with breast cancer
Source: Breast Cancer Res Treat. 2026 Mar 7;216(2):23. doi: 10.1007/s10549-026-07937-1 (PMC12967492; doi:10.1007/s10549-026-07937-1)
Supplement: Supplementary file 2 — Supplementary file2 (DOCX 16 KB) [file 10549_2026_7937_MOESM2_ESM.docx]

Supplementary Table 2: Demographic Differences Between Patients Who Died from T-DXd Related Infections Compared to Those Who Did Not

|  | Infection not Resulting in Death (n=26) | Infection Resulting in Death (n=6) | p-value |
| --- | --- | --- | --- |
| Age | 61 (46, 69) | 54 (50, 60) | 0.45 |
| BMI | 27 (23, 33) | 29 (26, 38) | 0.41 |
| Smoking | 7 (26.9%) | 1 (16.7%) | 1.00 |
| Diabetes | 5 (19.2%) | 1 (16.7%) | 1.00 |
| Cirrhosis | 4 (15.4%) | 1 (16.7%) | 1.00 |
| Chronic Pulmonary Diseases^1^ | 4 (15.4%) | 2 (33.3%) | 0.31 |
| Significant Corticosteroid Exposure During T-DXd Treatment^2^ | 5 (20.0%) | 1 (16.7%)^3^ | 1.00 |
| Lymphocyte Count at Treatment Initiation (cells/ µL) | 1,230 (880, 1,550) | 1,370 (680, 1,760) | 0.96 |
| Neutrophil Count at Treatment Initiation (cells/ µL) | 4,065 (2,780, 5,270) | 5,070 (2,870, 9,350) | 0.62 |
| Lymphocyte Count at Infection (cells/ µL) | 1,000 (590, 1,120) | 260 (150, 360) | 0.07 |
| Neutrophil Count at Infection (cells/ µL) | 3,130 (1,720, 7,360) | 985 (0, 4,960) | 0.12 |

^1^Chronic pulmonary disease: Includes chronic obstructive pulmonary disease, asthma, and interstitial lung disease

^2^Steroid equivalent of prednisone 20 mg for at least 7 days of consecutive treatment

^3^Was on significant corticosteroids at the time of infection
