# Supplementary material for: Comparing infectious risk of Trastuzumab-deruxtecan to Trastuzumab-emtansine in patients with breast cancer
Source: Breast Cancer Res Treat. 2026 Mar 7;216(2):23. doi: 10.1007/s10549-026-07937-1 (PMC12967492; doi:10.1007/s10549-026-07937-1)
Supplement: Supplementary file 3 — Supplementary file3 (DOCX 17 KB) [file 10549_2026_7937_MOESM3_ESM.docx]

Supplementary Table 3: Risk Factors for Mild and Severe Infections from T-DXd Treatment

|  | Mild (n=13) | Severe (n=19) | p-value |
| --- | --- | --- | --- |
| Age | 64 (51, 68) | 55 (46, 64) | 0.39 |
| BMI | 27 (26, 35) | 26 (22, 33) | 0.18 |
| Smoking | 3 (23.1%) | 5 (26.3%) | 1.00 |
| Diabetes | 3 (23.1%) | 3 (15.8%) | 0.67 |
| Cirrhosis | 2 (15.4%) | 3 (16.7%) | 1.00 |
| Chronic Pulmonary Diseases^1^ | 3 (23.1%) | 3 (16.7%) | 0.67 |
| Significant Corticosteroid Exposure During T-DXd Treatment^2^ | 2 (16.7 %) | 4 (21.1%)^3^ | 1.00 |
| Incidence of Hospitalization | 9 (69.2%) | 18 (94.7%) | 0.13 |
| Central Lines | 11 (84.6%) | 16 (84.2%) | 1.00 |
| Lymphocyte Count at Treatment Initiation (cells/ µL) | 1,310 (1,150, 1,640) | 1,110 (810, 1,550) | 0.13 |
| Neutrophil Count at Treatment Initiation (cells/ µL) | 3,830 (2,970, 4,730) | 4,130 (2,520, 7,370) | 0.73 |
| Lymphocyte Count at Infection (cells/ µL) | 1,080 (790, 1,280) | 455 (150, 1,000) | 0.005 |
| Neutrophil Count at Infection (cells/ µL) | 4,190 (2,410, 5,730) | 2,265 (800, 7,920) | 0.38 |

^1^Chronic pulmonary disease: Includes chronic obstructive pulmonary disease, asthma, and interstitial lung disease

^2^Steroid equivalent of prednisone 20 mg for at least 7 days of consecutive treatment

^3^Three of the 4 were exposed to significant corticosteroids at time of infection
